# Substantial LIB Anode Performance of Graphitic Carbon Nanoflakes Derived from Biomass Green-Tea Waste

**DOI:** 10.3390/nano9060871

**Published:** 2019-06-07

**Authors:** Sankar Sekar, Youngmin Lee, Deuk Young Kim, Sejoon Lee

**Affiliations:** 1 Department of Semiconductor Science, Dongguk University-Seoul, Seoul 04620, Korea; sanssekar@gmail.com (S.S.); ymlee@dongguk.edu (Y.L.); dykim@dongguk.edu (D.Y.K.); 2 Quantum-functional Semiconductor Research Center, Dongguk University-Seoul, Seoul 04620, Korea

**Keywords:** biomass, mesoporous graphitic carbon, anode, lithium-ion battery

## Abstract

Biomass-derived carbonaceous constituents constitute fascinating green technology for electrochemical energy-storage devices. In light of this, interconnected mesoporous graphitic carbon nanoflakes were synthesized by utilizing waste green-tea powders through the sequential steps of air-assisted carbonization, followed by potassium hydroxide activation and water treatment. Green-tea waste-derived graphitic carbon displays an interconnected network of aggregated mesoporous nanoflakes. When using the mesoporous graphitic carbon nanoflakes as an anode material for the lithium-ion battery, an initial capacity of ~706 mAh/g and a reversible discharge capacity of ~400 mAh/g are achieved. Furthermore, the device sustains a large coulombic efficiency up to 96% during 100 operation cycles under the applied current density of 0.1 A/g. These findings depict that the bio-generated mesoporous graphitic carbon nanoflakes could be effectively utilized as a high-quality anode material in lithium-ion battery devices.

## 1. Introduction

A continuous global demand for green energy technology encouraged tremendous efforts toward the evolution of renewable and environmentally friendly devices that can effectively convert and store efficient energy sources [1,2]. Among various energy devices, lithium-ion batteries (LIBs) are regarded as next-generation mobile electrical-energy-storage modules because of their high energy capacity and the excellent cyclic durability. The electrochemical performances of LIBs strongly rely on the choice of the electrode material; hence, exploiting a high-quality electrode source material is necessary to improve the energy-storage capacity of LIBs [3,4,5]. To date, several types of electrode materials (e.g., conductive polymers, transition metal oxides, carbonaceous materials, hydroxides, etc.) were investigated and proposed as feasible anode sources for LIBs [6,7,8,9,10,11]. Compared to other materials, carbonaceous constituents (e.g., activated carbon, carbon nanotubes, graphitic carbon, graphene, graphite, etc.) could become suitable candidates because of their high safety, high electrical conductivity, low cost, excellent electrochemical stability, high mechanical robustness, and outstanding electrolyte-ion accessibility [12,13,14]. In particular, porous graphitic carbon nanostructures emerged as a powerful candidate because they can provide a small diffusion pathway for ion transport, as well as a large active area for the electrochemical reaction [15,16]. In addition, biomass-derived porous graphitic carbon nanostructures are of ample interest due to their cost effectiveness, environmental friendliness, vast abundance, and fast regeneration. According to previous literature, it was reported that the biomass graphitic carbon nanostructures could be derived and synthesized from various natural resources such as cellulose [17], sucrose [18], natural cotton [19], garlic peel [20], wheat flour [21], *Sterculia scaphigera* [22], wheat stalk [23], and waste tea powders [24,25,26]. Among them, the green-tea waste is a cheap and abundant biomass source. The amount of world green-tea production in 2015 was 1.8 billion kilograms for consumption [25], and the green-tea waste contains 3.5–7% inorganic components, as well as 93.0–96.5% organic components [27,28]. In this regard, recently, we derived high-quality graphitic carbon nanoflakes from green-tea waste, and used them as an electrode source material for a high-performance supercapacitor [26]. Despite such great potential of green-tea-derived biomass graphitic carbon, its electrochemical performance as an LIB anode was rarely investigated except for a few previous studies [24,25].

Motivated by all the above, we derived the interconnected mesoporous graphitic carbon (IMP-GC) nanoflakes from waste green-tea powders, and characterized their astonishing material properties as an LIB anode material. The IMP-GC nanoflakes were synthesized via the following procedures of eco-friendly simple steps: air-assisted carbonization, followed by potassium hydroxide (KOH) activation and water treatment. The structural, microstructural, textural, and electrochemical characteristics of the synthesized IMP-GC nanoflakes were thoroughly examined in detail.

## 2. Experimental Details

### 2.1. IMP-GC Nanoflake Synthesis

**Figure 1:**
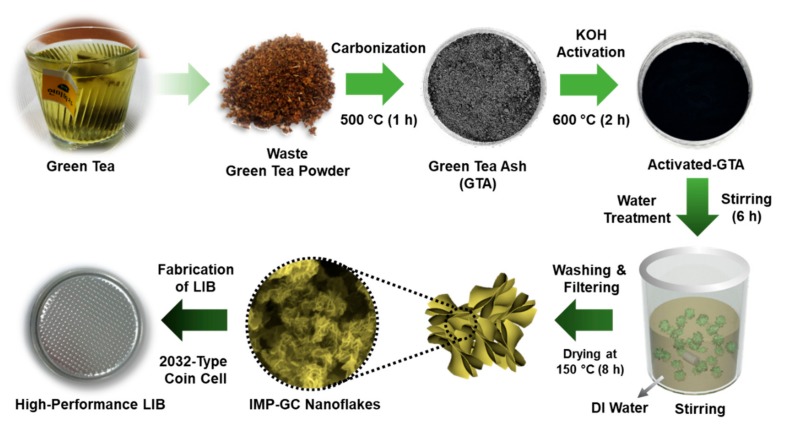
Schematic illustration of the synthesis procedures for the preparation of interconnected mesoporous graphitic carbon (IMP-GC) nanoflakes using a biomass carbonaceous resource derived from green-tea waste via potassium hydroxide (KOH) activation.

Figure 1 shows the experimental schemes for the derivation and the synthesis of the IMP-GC nanoflakes using waste green-tea powders through air-assisted carbonization, KOH activation, and water treatment. For this process, we used the waste green-tea powders (from green-tea bags) as a biomass carbonaceous resource. Firstly, the raw materials of the waste green-tea powders were transferred into the alumina crucible, and were carbonized in air at 500 °C for 60 min to accumulate their residues (i.e., green-tea ashes (GTAs)). Then, GTAs were mixed with KOH at a molar ratio of GTA/KOH = 1/4, and the GTA–KOH mixtures were activated in air at 600 °C for 120 min. Thereafter, the KOH-activated GTA products were treated with water by blending them in deionized water for 6 h. Here, the water treatment was aimed at enhancing the graphitization rate of the IMP-GC nanoflakes [26]. After filtering out the precipitates of potassium compounds, the obtained graphitic carbon products were cleaned again by deionized water, and were dried at 150 °C for 8 h in vacuum.

### 2.2. Measurements of Material Characteristics 

The crystallographic structure of IMP-GC was characterized through X-ray diffraction (XRD), and the graphitic nature of IMP-GC was analyzed by Raman scattering spectroscopy under green laser excitation (λ = 532 nm). The morphology and the microstructure of IMP-GC were monitored by scanning electron microscopy (SEM, Phillips, Eindhoven, The Netherlands) and transmission electron microscopy (TEM, JEOL USA Inc., Peabody, MA, USA), respectively. The textural pore characteristics were evaluated by nitrogen adsorption–desorption isotherm measurements at 77 K. 

### 2.3. Electrochemical Performance Measurements

The LIB performances (i.e., electrochemical properties of IMP-GC as an LIB anodic source) were assessed using a 2032-type coin cell. The anodic source of LIB was prepared by blending the IMP-GC nanoflakes (80%) in an *N*-methyl-2-pyrrolidinone solution. We here note that 10% polyvinylidene difluoride and 10% carbon black were also incorporated as additives for increasing the network conductivity of the IMP-GC nanoflakes. The prepared anodic source was then painted onto the current collector part (i.e., copper foil). After subsequent curing at 80 °C for 10 h, the electrode was assembled in an LIB coin cell under Ar atmosphere. The mass of the loaded electrode source material was ~0.82 mg. Here, the separator and the counter electrode were configured with a Celgard trilayer (PP/PE/PP) and a lithium foil, respectively. In addition, the electrolyte was composed of an LiPF_6_ solution (1 M) with a combination of dimethyl carbonate and ethylene carbonate (1:1). The cyclic voltammetry (CV) properties of the fabricated LIB device were examined at a scan rate (r_sc_) of 1 mV/s within the potential range of 0.01–3.0 V (vs. Li/Li^+^). The galvanostatic charge–discharge (GCD) characteristics were analyzed at 0.01–3.0 V by injecting the applied current (J_a_) of 100 mA/g. The electrochemical impedance spectrometry (EIS) measurements were conducted at frequencies of 0.01–100 kHz. 

## 3. Results and Discussion

**Figure 2:**
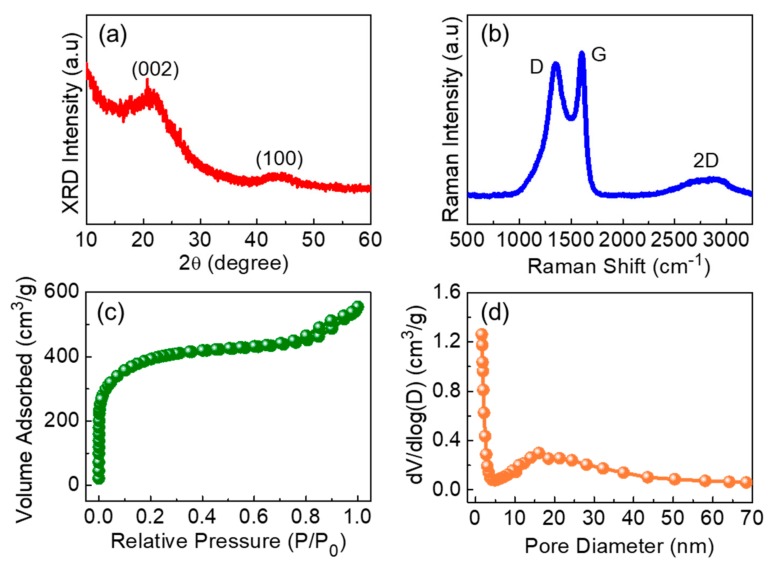
(**a**) X-ray diffraction (XRD) pattern, (**b**) Raman spectrum, (**c**) N_2_ adsorption–desorption isotherm characteristics, and (**d**) pore size distribution of the IMP-GC nanoflakes derived from waste green tea.

GTA-derived IMP-GC displays no distinct diffraction patterns except for (002) and (100) phases (Figure 2a). The broad peak at ~22° is a typical XRD pattern from the (002) crystallized carbon phase, and is indicative of the layered structure of multiple graphene sheets. The small peak of the (100) phase at ~44° arises from *sp*^2^-hybridized carbon with a honeycomb structure [9,29,30]. This represents that our IMP-GC nanoflakes possess a graphitic structure of carbon materials. The precise features of the IMP-GC nanoflakes can be further elucidated from their Raman scattering characteristics (Figure 2b). The sample exhibits three predominant peaks from D, G, and 2D bands, originating from the local vibration of disordered/defective features of graphite structures, the E_2g_ vibration mode in *sp^2^*-hybridized carbon, and the stacking of graphene sheets (i.e., signature of graphitic carbon), respectively [31,32]. Here, it is worth noting that the intensity ratio of D to G (i.e., I_D_/I_G_) is ~0.93. Since the magnitude of I_D_/I_G_ depicts a degree of graphitization in carbonaceous materials [33], one can conjecture that the biomass GTA-derived IMP-GC nanoflakes are highly graphitized. The total mass loss of the air-assisted green-tea powders (Figure S1, Supplementary Materials) is around 24.2%, demonstrating the high thermal stability of the materials [34].

The textural properties of IMP-GC were evaluated by the nitrogen adsorption–desorption isotherm measurements at 77 K. The IMP-GC nanoflakes reveal an isotherm characteristic of type I/IV adsorption–desorption with a type H4 hysteresis loop (Figure 2c), associated with both mesoporous and microporous structures [35,36]. Through the Brunauer–Emmett–Teller method, it was confirmed that the IMP-GC nanoflakes have a relatively large specific surface area (~1373 m^2^/g) and a great total pore volume (~0.4093 cm^3^/g). These are much greater than those of other green-tea-derived graphitic carbon nanostructures [24,25,26]. Figure 2d displays the pore characteristics of the IMP-GC nanoflakes. By means of the Barrett–Joyner–Halenda analysis, the average pore size and the pore surface area of the IMP-GC nanoflakes were calculated to be ~1.62 nm and ~479.52 m^2^/g, respectively. The microporous nature of the IMP-GC nanoflakes was also confirmed from the t-plot analysis (see Figure S1, Supplementary Materials). Specifically, the micropore volume and size of the IMP-GC nanoflakes were measured to be ~0.5066 cm^3^/g and ~0.72 nm, respectively, from the t-plot analysis. These specify that the IMP-GC nanoflakes are constructed with a micro/mesoporous structure. Both the large surface area and the micro/mesoporous structure render an electrolyte–electrode interfacial area huge enough to accumulate abundant charges and ions; hence, the electrochemical performances of IMP-GC as an LIB anode could be enhanced, as explained in detail later.

**Figure 3:**
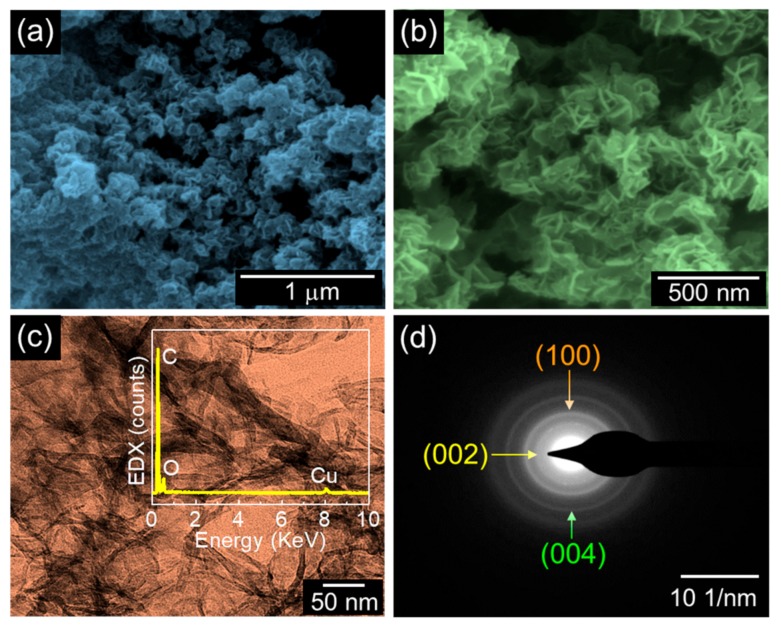
Morphological, microstructural, and compositional properties of the IMP-GC nanoflakes: (**a**) field-emission (FE)-SEM image; (**b**) zoomed-in FE-SEM image; (**c**) high-resolution TEM image with the in situ energy-dispersive X-ray (EDX) spectrum (inset); (**d**) selective-area electron diffraction pattern.

Figure 3a,b show the SEM images of the GTA-derived IMP-GC nanoflakes. The sample exhibits an interconnected network of the mesoporous GC nanoflakes with a width of about 6–10 nm. When the annealing temperature was increased from 600 to 900 °C, the samples showed an aggregated flake structure (see Figure S3, Supplementary Materials). The microstructure of the IMP-GC nanoflakes was further elucidated by TEM. As displayed in Figure 3c, the IMP-GC nanoflakes are composed of highly interconnected mesoporous activated carbon nanoflakes. From the in situ energy-dispersive X-ray (EDX) measurement, the IMP-GC nanoflakes were ratified to consist of a major component of C (inset of Figure 3c). Small amounts of Cu and O are thought to arise from the TEM grid. The selective-area electron diffraction patterns (i.e., ring patterns) pronounce that the IMP-GC nanoflakes are well graphitized, as confirmed from the XRD and Raman analyses.

**Figure 4:**
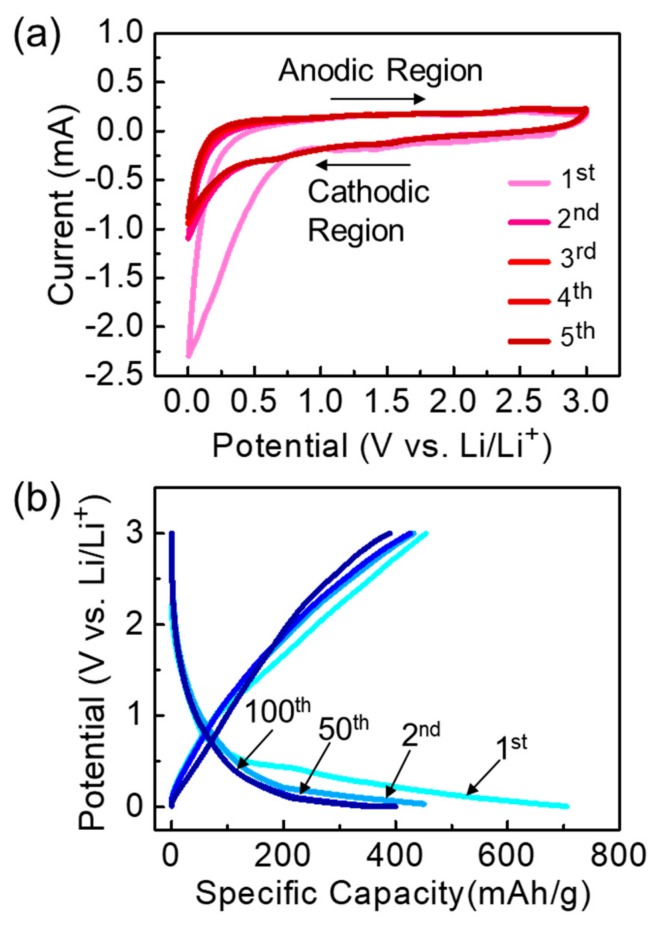
(**a**) Cyclic voltammetry (CV) curves measured under r_sc_ of 0.1 mV/s, and (**b**) galvanostatic charge–discharge (GCD) curves measured under J_a_ of 0.1 A/g for the lithium ion battery (LIB) device with the anode material of IMP-GC nanoflakes.

After confirming the effective synthesis of biomass IMP-GC nanoflakes using green-tea waste, we assessed their electrochemical characteristics as an LIB anode material. Figure 4a displays the CV curves of the fabricated LIB sample measured under r_sc_ = 0.1 mV/s within 0–3.0 V (vs. Li/Li^+^). The first cyclic CV curve shows a wide reduction in the cathodic region, which is indicative of both the formation of the solid electrolyte interface (SEI) layer onto the IMP-GC surface and the decomposition of the electrolyte [37,38,39]. Such a wide reduction disappears during consecutive cycles, while the CV curves maintain an almost identical area during all subsequent cycles. This represents the high stability of the IMP-GC structure. Figure 4b represents the first, second, 50th, and 100th GCD profiles measured at constant J_a_ of 0.1 A/g. Due to both the SEI formation and the other-side reaction [37,40,41], the LIB device reveals two plateaus at ~0.5 and ~0.75 V during the first discharge cycle. In subsequent cycles, these two plateaus disappear because of the high stability of IMP-GC. The initial charge and discharge capacity values reach ~455 and ~706 mAh/g, respectively. Accordingly, the initial coulombic efficiency of the IMP-GC electrode is ~64.4%. The high irreversible capacity is observable for the first GCD cycle because of the irreversible Li^+^ intercalation into the particular IMP-GC position and/or the creation of the SEI layer [42,43]. Since the SEI layer suppresses the electrolyte decomposition on the carbon electrode, the shapes of the GCD profiles become distinctive again and again for the following cycles. In addition, the reversible capacities are stabilized while the irreversible capacities disappear. From the GCD profiles, it was also confirmed that the discharge capacity of IMP-GC is comparable to and even higher than that of other biomass graphitic and activated carbon nanostructures [20,21,22,23,24,25,43,44,45,46,47,48,49,50,51] (see Table 1). 

**Figure 5:**
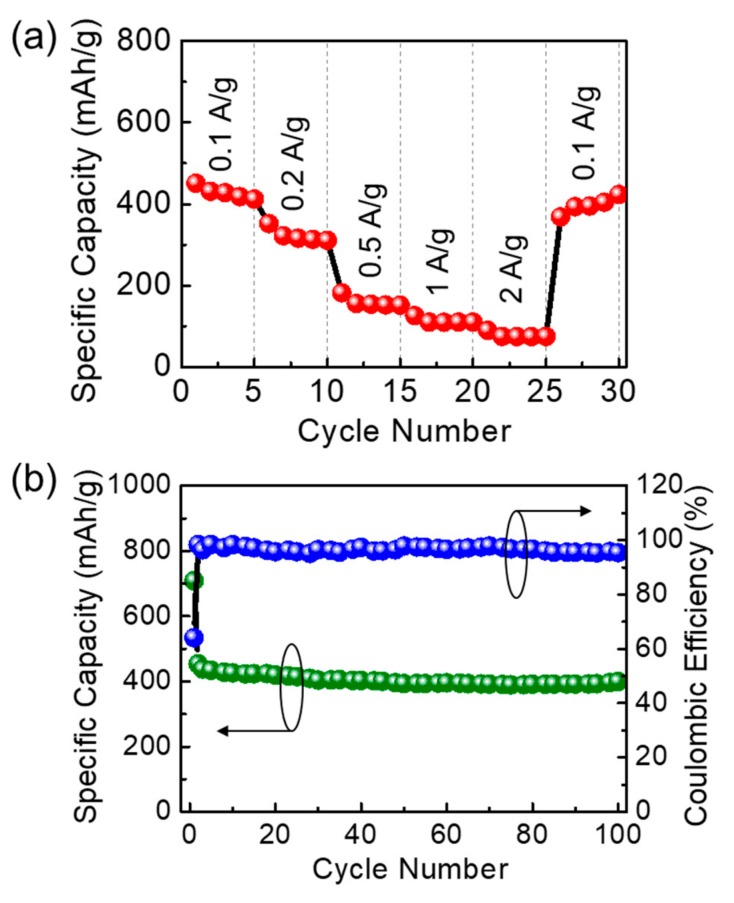
(**a**) Rate performance at various J_a_, and (**b**) cyclic performance with its corresponding coulombic efficiency at J_a_ of 0.1 A/g for the LIB device with the anode material of IMP-GC nanoflakes.

Next, we examined the rate capability of the LIB sample by changing J_a_ from 100 to 2000 mA/g. As can be seen from Figure 5a, the device exhibits the discharge capacities of 454, 352, 183, 125, and 90 mAh/g at J_a_ of 0.1, 0.2, 0.5, 1, and 2 A/g, respectively. Owing to the high surface area of uniform pores in IMP-GC, more than 20% of the specific discharge capacity is retained even after applying high J_a_ up to 2 A/g. When returning back to J_a_ = 0.1 A/g, the discharge capacity is dramatically recovered to the original value. This demonstrates the excellent reversibility of the IMP-GC electrode. In addition, the IMP-GC nanoflakes also exhibit a good cyclic performance as an LIB anode. During 100 cycles under J_a_ = 0.1 A/g, the device sustains its large coulombic efficiency up to 96% (Figure 5b). However, the discharge capacity was slightly decreased by ~12% (i.e., from ~454 to ~400 mAh/g), presumably because of the SEI decomposition via continuous insertion and extraction of Li ions for multiple discharge–charge cycles [52]. 

**Figure 6:**
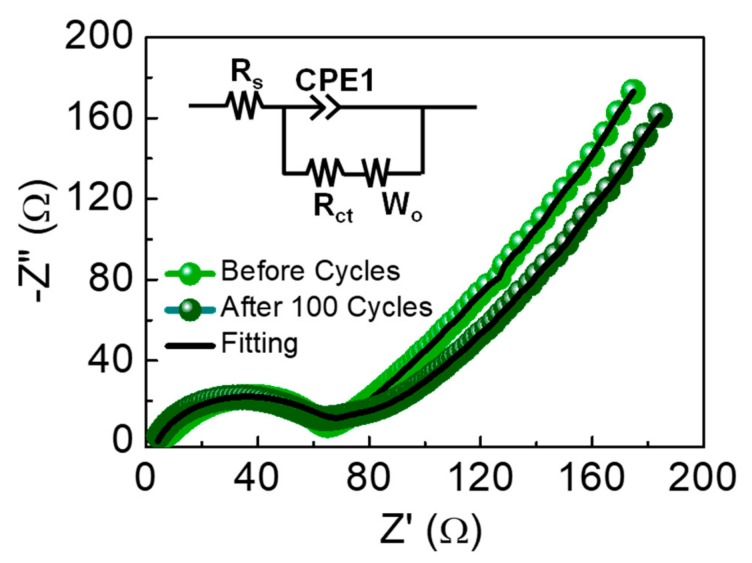
Nyquist plots before and after 100 charge–discharge cycles at J_a_ = 0.1 A/g and the equivalent circuit (inset) for the LIB device with the anode material of IMP-GC nanoflakes.

In energy-storage devices, the charge transfer resistance (R_ct_) is one of the most key parameters that represent the interface property between the electrolyte and the electrode. To determine R_ct_, we measured the EIS characteristics of the LIB device. Figure 6 displays the Nyquist plots of the sample measured before and after the cyclic process at J_a_ = 0.1 A/g. The Nyquist plots include two distinguishable features of a semicircle and a long tail. Since the former at a higher-frequency region and the latter at a lower-frequency region depict R_ct_ and Warburg impedance (W_o_, i.e., degree of Li ion diffusion into the electrode) [23], respectively, one can extract the R_ct_ value by analyzing the equivalent circuit for the fabricated LIB device (inset of Figure 6). Before the cyclic process, the device revealed a relatively lower value of R_ct_ ~60 Ω; then, the magnitude of R_ct_ was further decreased to ~54 Ω after the cyclic process because of the SEI formation. From all the above results, we can conclude that the biomass GTA-derived IMP-GC nanoflakes could effectively play a substantial role as an anodic source material for high-performance LIBs.

## 4. Conclusions

The IMP-GC nanoflakes were successfully synthesized using biomass carbonaceous resources of GTAs that were obtained from waste green-tea powders. From the morphological and microstructural characteristics, the GTA-derived IMP-GC nanoflakes were confirmed to form an interconnected network of mesoporous nanoflakes having a vast specific surface area (i.e., ~1373 m^2^/g). The IMP-GC nanoflakes were also shown to play a substantial role as an LIB anode material. Specifically, the LIB device with the anode of IMP-GC nanoflakes revealed an outstanding rate performance (i.e., Li ion storage up to ~400 mAh/g with a coulombic efficiency of ~96% during 100 cycles under J_a_ = 0.1 A/g). Consequently, the biomass GTA-derived IMP-GC nanoflakes hold promise for low-cost green technology, particularly aimed at designing high-performance electrochemical energy-storage devices.

## Figures and Tables

**Table 1:** Comparison of electrochemical performances for various biomass-derived carbon nanostructures used as lithium ion battery (LIB) anode materials.

Biomass Resource	Measurement Condition	Initial Capacity (mAh/g)	Capacity Retention (mAh/g)	Reference
Waste green tea	0.1 A/g	706	400 at 0.1 A/g after 100 cycles	This work
Garlic peel	0.1 A/g	551	540 at 0.1 A/g after 100 cycles	20
Wheat flour	1 C	728	217 at 1 C after 100 cycles	21
*Sterculia scaphigera*	0.1 C	1539	423 at 0.1 C after 100 cycles	22
Wheat stalk	0.1 C	502	~140 at 10 C after 3000 cycles	23
Green tea leave	0.1 C	530	~450 at 0.1 C after 50 cycles	24
Waste green tea	0.1 C	869	479 at 0.2 C after 200 cycles	25
Walnut shell	0.1 A/g	150	150 at 0.1 A/g after 100 cycles	43
Peanut shell	1 A/g	761	314 at 1 A/g after 400 cycles	44
Sugar	0.1 A/g	477	-	45
Cherry stones	0.1 C	790	210 at 0.1 C after 100 cycles	46
Orange peel	1 A/g	878	301 at 1 A/g after 100 cycles	47
Petroleum coke	0.1 C	320	293 at 0.1 C after 300 cycles	48
Coffee waste	0.1 A/g	359	262 at 0.1 A/g after 100 cycles	49
Alginic acid	0.7 C	420	80 at 45 C after 1500 cycles	50
Olive stones	0.2 C	615	170 at 0.2 C after 100 cycles	51

Note: 1 C = 372 mA/g.

## Supplementary Materials

The following are available online at https://www.mdpi.com/2079-4991/9/6/871/s1, Figure S1: Thermogravimetric analysis of the green tea powder ash; Figure S2: T-plot of the IMP-GC nanoflakes; Figure S3: Field-emission SEM images of the IMP-GC nanoflakes activated at (a) 700 °C, (b) 800 °C, and (c) 900 °C.
