# Supplementary material for: Substantial LIB Anode Performance of Graphitic Carbon Nanoflakes Derived from Biomass Green-Tea Waste
Source: Nanomaterials (Basel). 2019 Jun 7;9(6):871. doi: 10.3390/nano9060871 (PMC6631619; doi:10.3390/nano9060871)
Supplement: Supplementary file 1 [file nanomaterials-09-00871-s001.pdf]

Supplementary Materials

Substantial LIB Anode Performance of Graphitic Carbon Nanoflakes Derived from Biomass Green-Tea Waste

Sankar Sekar,[1,2] Youngmin Lee,[1,2] Deuk Young Kim,[1,2] and Sejoon Lee[1,2,*]

[1] *Department of Semiconductor Science, Dongguk University-Seoul, Seoul 04620, Republic of Korea*

[2] *Quantum-functional Semiconductor Research Center, Dongguk University-Seoul, Seoul 04620, Republic of Korea*

[*] **Corresponding Author:** E-mail: sejoon@dongguk.edu (S. Lee)

Tel: +82-2-2260-3946 / Fax: +82-2-2260-3945 / ORCID ID: 0000-0002-4548-7436

■ Thermogravimetric (TG) Analysis of Green Tea Powder Ash

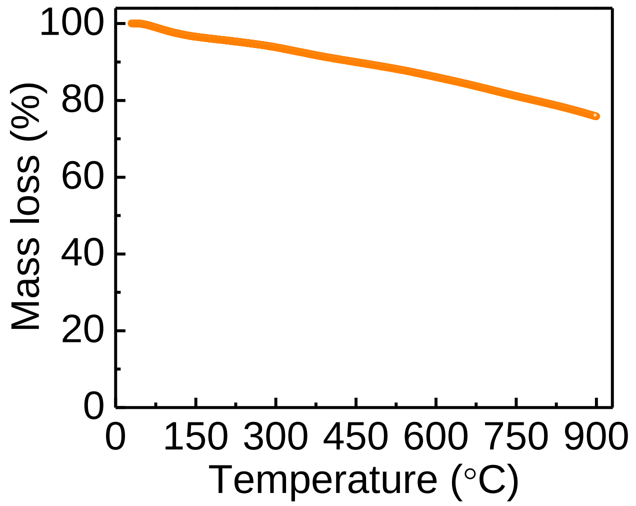

Figure S1. Thermogravimetric analysis of the green tea powder ash.

The TG analysis was performed to study the desorption-calcination of the green tea powder ash (Fig. S1). The first mass loss around 40 - 200°C can be ascribed to the existence of physisorbed and unbounded water. At temperature from 300 to 600 °C, a progressive mass loss occurs in the green tea powder ash, which demonstrates the thermal decomposition of the organic materials in the green tea powder ash [S1,S2]. The total mass loss of the air assisted green-tea powder is around 24.2%, and this represents the high thermal stability of the materials.

■ T-plot of the IMP-GC nanoflakes

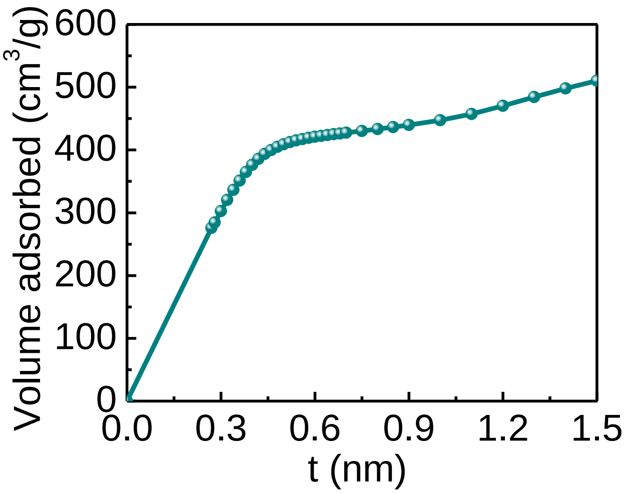

Figure S2. T-plot of the IMP-GC nanoflakes.

■ T-plot of the IMP-GC nanoflakes

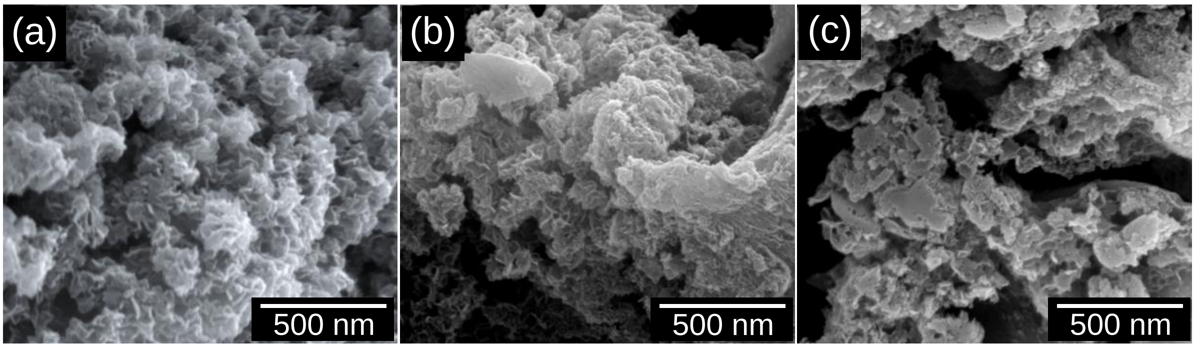

Figure S3. Field-emission SEM images of the IMP-GC nanoflakes activated (a) 700 °C, (b) 800 °C, and (c) 900 °C.

References

S1. Bazan, A.; Nowicki, P.; Półrolniczak, P.; Pietrzak, R. J. J. o. T. A.; Calorimetry, Thermal analysis of activated carbon obtained from residue after supercritical extraction of hops. *Journal of Thermal Analysis and Calorimetry* **2016**, 125, 1199-1204.

S2. Luna-Lama, F.; Rodríguez-Padrón, D.; Puente-Santiago, A. R.; Muñoz-Batista, M. J.; Caballero, A.; Balu, A. M.; Romero, A. A.; Luque, R., Non-porous carbonaceous materials derived from coffee waste grounds as highly sustainable anodes for lithium-ion batteries. *Journal of Cleaner Production* **2019**, 207, 411-417.
